# Novel insights into gene expression regulation during meiosis revealed by translation elongation dynamics

**DOI:** 10.1038/s41540-019-0089-0

**Published:** 2019-04-04

**Authors:** Renana Sabi, Tamir Tuller

**Affiliations:** 1 Department of Biomedical Engineering, Tel Aviv University, Tel Aviv, Israel; 2 The Sagol School of Neuroscience, Tel-Aviv University, Tel-Aviv, Israel

**Keywords:** Evolution, Computational biology and bioinformatics, Systems biology, Mathematics and computing, Genetics

## Abstract

The ability to dynamically control mRNA translation has a great impact on many intracellular processes. Whereas it is believed that translational control in eukaryotes occurs mainly at initiation, the condition-specific changes at the elongation level and their potential regulatory role remain unclear. Using computational approaches applied to ribosome profiling data, we show that elongation rate is dynamic and can change considerably during the yeast meiosis to facilitate the selective translation of stage-specific transcripts. We observed unique elongation changes during meiosis II, including a global inhibition of translation elongation at the onset of anaphase II accompanied by a sharp shift toward increased elongation for genes required at this meiotic stage. We also show that ribosomal proteins counteract the global decreased elongation by maintaining high initiation rates. Our findings provide new insights into gene expression regulation during meiosis and demonstrate that codon usage evolved, among others, to optimize timely translation.

## Translation elongation regulation and dynamics during meiosis

During translation, the ribosome is attached to an mRNA and decodes it into a protein. Since translation consumes most of the intracellular energy, it is tightly regulated. It is believed that translation is mainly controlled by the rate at which ribosomes enter the mRNA. However, it remains unclear whether the progression rate of the ribosomes along the mRNA (i.e. the elongation rate) can dynamically change and regulate translation. By computationally analyzing experimental data of the ribosome speeds during yeast meiosis, we show that these elongation rates can change dramatically. Our analyses suggest that these changes in the elongation rates of various genes are shaped by evolution according to their function, to improve organismal fitness.

## Introduction

For over decades, most of the gene expression studies were based on transcript levels measured by techniques such as microarrays and RNA-seq.^1^ The development of ribosome profiling in 2009^2^ allowed for the first time a high-resolution view into in vivo translation on a large scale. In the past few years, ribosome profiling has rapidly become a widely used tool for studying messenger RNA (mRNA) translation, yielding an increased number of studies on gene expression regulation at the translational level.^2–11^

In the context of translation regulation, however, a major focus is dedicated to the initiation phase and little is known about the unique role of the elongation phase. Specifically, whereas translation initiation is believed to be the major regulatory phase that dictates translation rates, translation elongation is rather assumed to occur at fixed rates and thus, not to play a role in a condition-specific translational control.^12–17^ Nevertheless, the idea that elongation rates can change under different conditions has been previously proposed. For example, simulation of translation based on the ribosome flow model in *S. cerevisiae* revealed that changes in the tRNA pool due to different levels of available glucose in the media lead to changes in the decoding rate of different codons.^18^ Another study by Frenkel- Morgenstern et al.,^19^ have suggested that cell cycle-regulated genes may use different synonymous codons to adjust their adaptation to the varying tRNA pool during the cell cycle; raising the prospect that elongation rates can vary in a functional manner. However, there is currently no direct, in vivo evidence regarding codon-specific changes in the typical decoding rates along multiple conditions.

A useful experiment for studying in vivo translation under different conditions is ribosome profiling. However, this approach provides information on the translation process as a whole, without isolating the specific contribution of the translation elongation stage from other gene expression aspects. Specifically, during the processing of ribosome profiling data, the positions of translating ribosomes are mapped over the entire transcriptome, producing transcript-specific ribosomal footprint-count profiles. Ideally, each profile is expected to provide a full picture of the decoding time at each position along the transcript.^2^ In practice, however, the generated read count (RC) is a superposition of transcript levels, initiation rates, elongation rates, noise and experimental biases.^20–22^ In addition, ribosomal footprints are mapped only to a very partial subset of codon positions mainly occurring within highly expressed genes,^21–23^ thus, restricting the ability to analyze all genes including very lowly expressed ones.

In this work, we analyze for the first time, the *elongation* rates of all *S. cerevisiae* genes in multiple time points/conditions during sporulation, based on the estimation of the Mean of the Typical codon Decoding Rate (MTDR).^23,24^ Using a mathematical and statistical model applied to ribosome profiling data, the MTDR extracts the specific component related to the elongation phase of translation, estimating the typical elongation rates of each codon.

To investigate translation elongation rates under different cellular conditions, we analyzed MTDR calculated based on ribosome profiling data sampled throughout meiosis in yeast.^4^

Meiosis is a conserved specialized form of cellular division, essential for sexual reproduction in nearly all eukaryotes. Particularly, it is of great importance in the production of genetic diversity and in the maintenance of a correct number of chromosomes in the progeny (reviewed in^25^). In single-celled budding yeast, *Saccharomyces cerevisiae*, meiosis is part of the process of sporulation which is initiated by transferring diploid yeast cells onto a nutritionally unbalanced medium. Starved for nitrogen and carbon, a diploid yeast cell undergoes a two-step nuclear division process, producing four haploid genetically distinct daughter cells. These products are packaged into spores within which they enter a dormant stationary phase. To enable a rapid response to the wide range of environmental changes that occur during meiotic sporulation, a tight control over gene expression is acting at both, the transcriptional^26,27^ and post-transcriptional levels.^4,28–30^ Specifically, a major study by Brar and co-workers has demonstrated a pervasive and dynamic translational control during the yeast meiotic sporulation program using ribosome profiling experiments on sporulating *S. cerevisiae* cells.^4^ Here, we applied the MTDR approach to the ribosome profiling data generated by Brar et al. to study the unique and dynamic role of translation elongation in translational control during meiosis. This work presents for the first time, a genome-wide study of translation elongation in multiple conditions based on the analysis of in vivo translation.

## Results

### Estimating translation elongation rates from ribosome profiling data

**Fig. 1:**
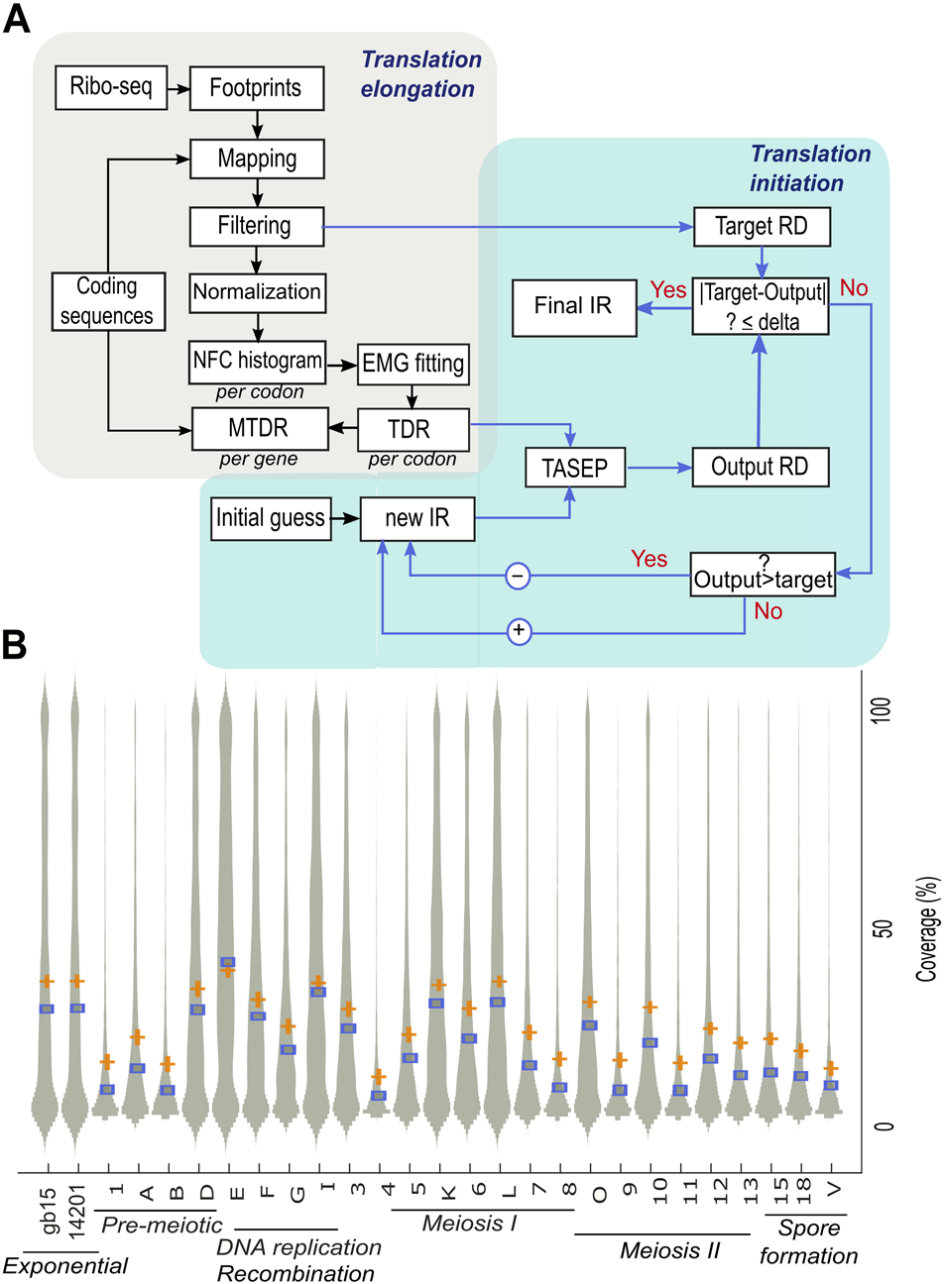
**a** Flow chart describing research approach: Ribo-Seq footprints at multiple time points were retrieved and mapped to the transcriptome. Positional RC profiles were generated and filtered. Each RC profile was normalized by its average RC, producing a transcript-specific NFC profile. For each codon, NFC values were collected from all NFC profiles, and per-codon histograms were generated. Using the MLE criterion, the histograms were fitted to an exponentially modified gaussian (EMG) distribution and the typical decoding rate of the codon was determined by the µ parameter. Finally, the MTDR of each gene was calculated by the mean of the typical decoding rates of its codons. Initiation rates were inferred using an optimization approach based on the totally asymmetric exclusion process (TASEP) model. The per-codon TDR and the ribosomal density were calculated based on the filtered ribo-seq profiles and ribosome occupancy data (details in the Methods). mRNA levels were calculated based on RNA-seq experiments performed at multiple time points along the yeast meiotic sporulation program.^4^ **b** Coverage of the RC profiles are shown on a violin plot. Values correspond to the raw sequencing data produced by Ribo-seq. Time points labels appear as in.^4^ Median values are denoted by blue squares and mean values by orange “+“ symbol

Our analyses rely on the separation of translational data obtained from ribosome profiling into elongation and initiation (general description of the approach is described in Fig. 1a). To this end, we utilize the MTDR, a novel estimation of translation elongation efficiency based on the analysis of ribosome profiling data.^23^ MTDR calculation consists of the following major steps: (1) generating codon-specific histograms of ribo-seq RC; (2) fitting each histogram into an exponentially modified gaussian, a superposition of a normal distribution and a negative exponential distribution; (3) using the maximum-likelihood estimation (MLE) to infer the mean of each normal distribution, which represents the mean of the typical decoding rate of each codon. The first stage of the MTDR calculation seeks to learn the footprint count distribution of each codon. To this end, RC profiles with a low coverage are filtered, and the remaining are chosen to consist a reliable reference set of profiles (distribution of the coverage is visualized in Fig. 1b). Then, each profile within the set is normalized by its average RC, enabling the comparison of RC originated from genes with different mRNA levels and initiation rates. In the next step, per-codon histograms are generated by going over each normalized footprint count (NFC) profile and collecting the NFC values in all occurrences of the codon. These NFC histograms are expected to represent the distribution of the decoding time of each codon and they resemble a log-normal distribution which is shaped as a normal distribution with a skewed right tail. It has been shown in^24^ that simulating translation without considering ribosomal pauses yields codon decoding rates that are normally distributed, suggesting that the right tail represents non-typical decoding rates; these can stem from either biases, traffic jams or extreme pauses. Thus, the second major stage aims at separating *typical* NFC values from *non-typical*, extreme values. This separation is achieved by decomposing the distribution into two components: a normal distribution corresponding to the typical decoding rate (TDR), and a negative exponential distribution corresponding to non-typical decoding rates (Methods).

In the final stage, the *typical* elongation time of each codon is estimated by the mean of the normal distribution component. Once all 61 codon decoding rates are inferred, per-gene MTDR is calculated for all genes by the geometric mean of the TDR of its codons.

### Fluctuations in the typical decoding rates throughout sporulation peaking at meiosis II

**Fig. 2:**
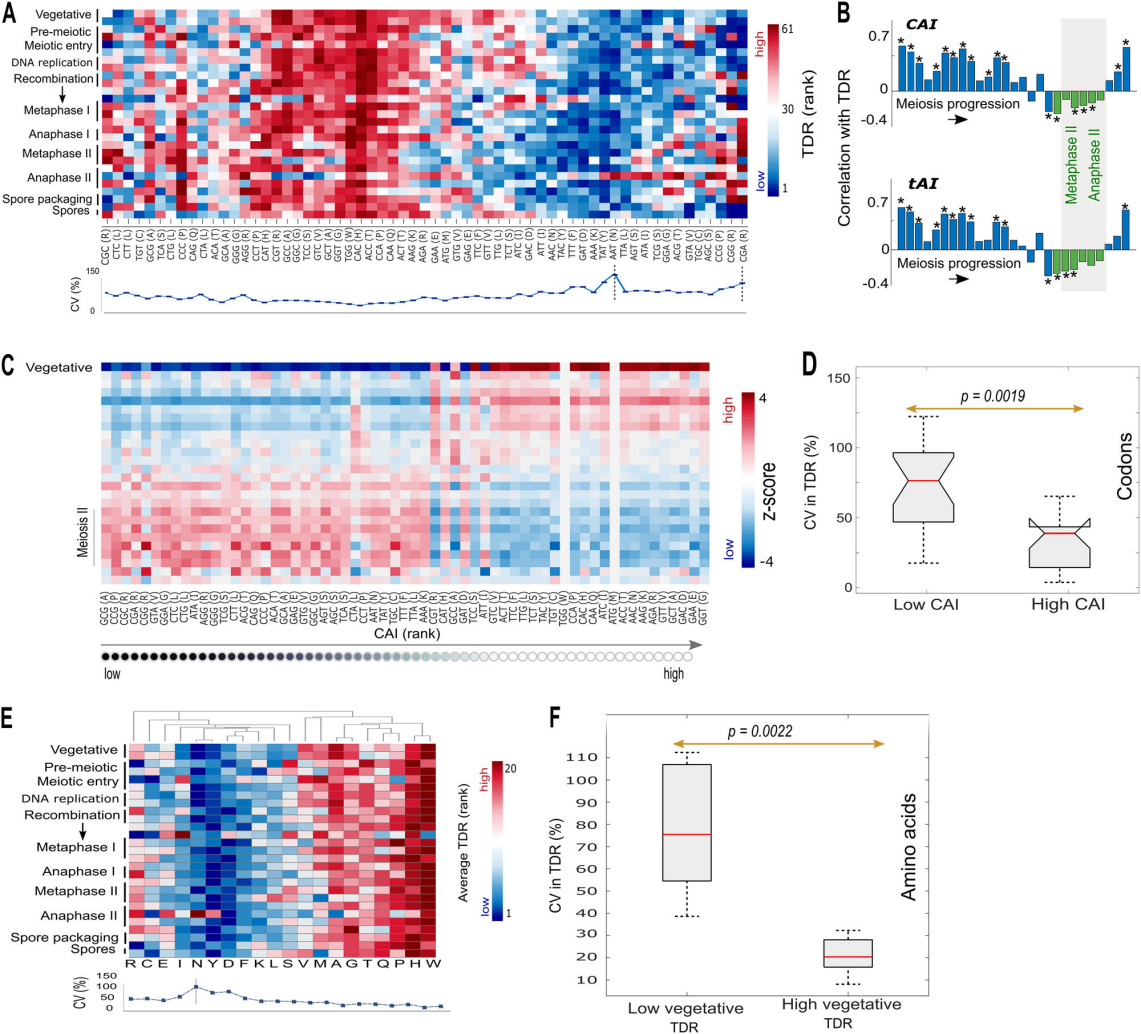
**a** Changes in codon decoding time during meiosis. Rows represent time points along sporulation and columns represent codons. To enable the comparison of TDR calculated at different time points, TDR are ranked at each time point. Blue and red entries correspond to low and high decoding rate, respectively. Codons are ordered according to a hierarchical clustering based on Euclidean distance. Below is the CV (%) corresponding to the relative variability in the TDR rank of each codon (“Methods” section). **b** Spearman’s rank correlations of codons’ TDR with CAI (top) and tAI (bottom) at each time point. Bars fall within meiosis II are colored in green and the area is shaded in gray. Significant correlations (*p* < 0.05) are designated by asterisks. **c**. The per-codon ratio between the total frequency of occurrences in all transcripts at vegetative growth, and at each other time point (Methods). Ratios are standardized per codon by the average over the time points. **d** Distribution of the CV in TDR of codons with the highest and lowest CAI; Methods). Medians are marked by horizontal red lines. P-value corresponding to the statistical difference between the medians is denoted. **e** Changes in the average TDR per-amino acid during meiosis. Rows represent time points along sporulation and columns represent amino acids. A dendrogram of the hierarchical clustering of the amino acids based on their average TDR pattern is shown above**. f** Distribution of the CV in TDR of amino acids with slow and high TDR at the vegetative growth time point. Medians are marked by horizontal red lines. P-value corresponding to the statistical difference between the medians is denoted. P-value remained significant after sampling equal number of RC for each codon (Supplementary Figure 2, Methods section)

Calculations of TDR for all 61 coding codons at 27 time points along meiotic sporulation revealed prominent fluctuations in the typical decoding rates during meiosis (Fig. 2a). The relative variability in the TDR during meiosis quantified using the coefficient of variation (CV), turned out to be codon-specific, varying from a minimum of 7% for the histidine codon, CAC, to 114.88% for the asparagine codon, AAT (Fig. 2a, bottom). Particularly, AAT (asparagine) and CGA (arginine) showed the highest variability amongst all codons. However, whereas the TDR of CGA showed fluctuations during most time points, the high CV of AAT was driven almost entirely by a dominant change at the onset of anaphase II. With respect to rank changes, the most dramatic shift was observed for two Arginine codons, CGA and CGG, which changed their rank from the lowest to the highest at least at one time point during meiosis. Intriguingly, both are known to be rare codons characterized by extremely low decoding rates under normal vegetative growth (Methods). The TDR rank of CGG, for example, had increased from the lowest rank at the vegetative growth phase, to the highest possible TDR rank at the onset of Metaphase I. A table summarizes all calculated TDR of the 61 codons at each of the 27 analyzed time points is provided as Supplementary Table 1.

Genomic/static measures of codon usage bias such as the tRNA Adaptation Index (tAI)^31^ and the Codon Adaptation Index (CAI)^32^ (Supplementary Tables 2-3) turned out to be positively correlated with the TDR of codons at most time points of meiotic sporulation. However, in 8 of the 27 analyzed time points, tAI and CAI exhibited negative Spearman’s rank correlations with decoding rates, implying that TDR cannot be trivially explained by codon usage bias. Prominently, the negative correlations were observed along two sequential meiotic stages, the metaphase II and the anaphase II (Fig. 2b).

Aiming at further understanding the factors affecting the observed decoding rates, we quantified the demand of each codon by its frequency in all presented mRNA at a given time point (Methods, Supplementary Table 4). Clearly, it can be observed that rare codons (in the genome) are significantly more overrepresented in the mRNAs presented in the cell during meiosis II, indicating an increased demand for these codons (Fig. 2c). Alternatively, frequent codons (in the genome) seem to rather be underrepresented at these stages (Fig. 2c).

To test whether the extent of fluctuations in TDR during meiosis is associated with codon usage bias, we compared the CV in TDR of rare and frequent codons (lowest and highest CAI, respectively; Methods) using a right-tailed Wilcoxon rank sum test. The group of rare codons turned out to be the least homogenous in this manner (median CV of 76.7% compared to 40.15% for the ‘frequent’ group, *p* = 2.34·10^−5^, Fig. 2d). Medians remained significantly different also after controlling for the different number of NFC values sampled for each codon (Methods, Supplementary Figure 1).

Seeking to determine whether changes observed at the codon level are specifically linked to changes at the amino acid level, for each amino acid at each time point, we calculated the mean decoding rate over its synonymous codons such that each codon contributes to the average TDR according to its genomic frequency (Methods). Although the Pearson correlation between the TDR pattern of codons and the TDR pattern of their corresponding amino acids was high (*r*^2^ > 0.67, *p* < 10^−323^, not including amino acids with only one codon), the TDR pattern observed at the amino acid level was generally more homogenous than the one observed at the codon level (Fig. 2e). However, asparagine (N) showed prominently high variability (CV of 108.1%). The most extreme shift in the average TDR was observed for asparagine (N) and arginine (R), who changed their rank from the top decile to the bottom at least at one time point during meiosis. In fact, asparagine and arginine are encoded by AAT and CGA respectively, which turned out to be the most variable codons.

As nitrogen and carbon are crucial components of all amino acids that become limited during sporulation,^33^ we speculated that the average TDR of amino acids which were already limited prior to meiosis, would be the most affected. Thus, we compared the relative change in the average TDR (in terms of CV) of the five amino acids with the highest TDR rank in the vegetative growth time points and the and the five amino acids with the lowest vegetative rank. Strikingly, the CV of the amino acids with the highest vegetative rank was higher (*p* = 0.0022, Fig. 2f).

### Dynamics in the elongation rates of genes during meiosis is related to their function

**Fig. 3:**
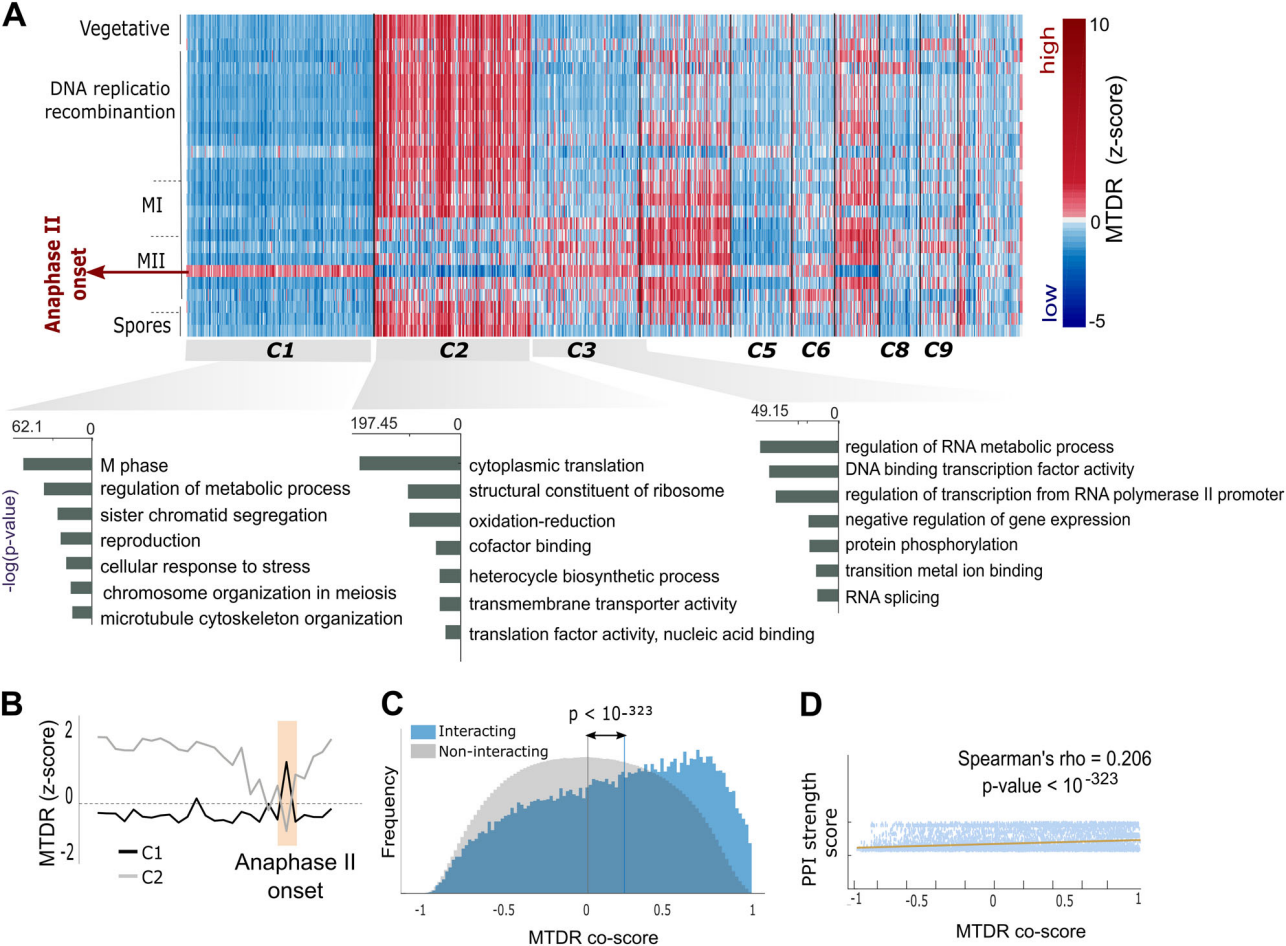
**a** Unsupervised clustering of the genes by the relative changes in MTDR along meiosis. MTDR were standardized to bring all time points to the same average so that z-scores represent the distance from the time point-average in terms of standard deviation. For each time point, z-scores greater/lower than the time point-average are shown in red/blue, respectively. Clusters are ordered by their size, from the largest to the smallest (from left to right). Meiotic stages are labeled to the left of the corresponding time points (rows). Top gene annotations enriched within C1, C2 and C3 are listed to the right, sorted based on their p-values (from the most significantly enriched to the less). **b** Mean MTDR values along meiosis for clusters C1 (black) and C2 (gray). The sharp opposite pattern observed at the onset of anaphase II is shaded in orange. **c** Distributions of MTDR co-scores for genes significantly associated with PPI (blue) versus genes of non-interacting proteins (gray). The *p-*value represents the difference between the medians of the two distributions. **d** Spearman’s rank correlation between MTDR co-score and PPI strength score. The correlation is based 21,638,331 points (corresponding to all possible pairs of the analyzed genes. Linear fitting of the points is denoted by an orange line

Given that MTDR represents an estimation of the elongation rate of codons, our analysis implies that the elongation rates of individual genes might be selectively affected during the different developmental stages of meiosis. To investigate this, we calculated MTDR for all *S. cerevisiae* genes in all 27 analyzed time points (Supplementary Table 6). To enable the comparison of different time points, all MTDR values were normalized for the time point and z-scores were obtained for each gene (Methods). Unsupervised clustering of the genes revealed 21 clusters, 7 of them turned out to emerge from functionally related groups (Fig. 3a).

Whereas different statistical measures based only on RC profiles require sufficient coverage, MTDR can be calculated for any input sequence, regardless of the depth of its RC profile coverage. Specifically, our analysis included all 6,579 budding yeast genes (excluding paralogous; Methods). Considering that, it is specifically important to emphasize clusters with very lowly expressed genes that cannot be captured by RC-based analysis. As can be prominently visualized in Fig. 3a, for example, we identified a large cluster of 1,497 genes (C1), all translated with very low elongation rates at most stages of meiosis (corresponding average RC coverage varies between 5% and 35%, depends on the time point). Interestingly, C1 turned out to be significantly enriched for genes involved in sister-chromatid segregation, precisely coherent with its sharp time point-specific shift toward increased elongation rate at the onset of anaphase II (Fig. 3b). The possible functional effect of the elongation rates of the C1 genes on the translational status, was further examined using whole cell simulation of translation that includes all the mRNA and ribosomes in the *S. cerevisiae* cell. Specifically, translation at the anaphase II onset was simulated twice: first, with the original genome and second, with a randomized version of the genome in which the codon composition of the C1 genes was randomly generated according to the genomic codon usage bias (Methods). Changing the synonymous codons in C1 resulted in a decrease in both, the pool of free ribosomes and the average translation rates (Supplementary Figure 3), which are expected to have very significant effect on the fitness of the organism.

Another prominent cluster of similar size (1,228 genes) but fundamentally different MTDR pattern, was C2 (Fig. 3a). In contrast to C1, C2 was mainly composed of highly expressed genes, translated with high elongation efficiency during most time points of meiosis. Consistently, functional enrichment analysis revealed that C2 is most significantly enriched for cytoplasmic translation (*p* = 1.7·10^−86^), and other housekeeping functions such as oxidation-reduction process (*p* = 3.3·10^−44^). Considerably low elongation rates of C2 genes were observed at the late phase of anaphase I, the middle phase of metaphase II and most prominently, at the onset of anaphase II (Fig. 3b). Strikingly, C3 showed an opposite pattern, presenting almost a perfect mirror-image of C2 (Fig. 3a). Most of the enriched annotations in C3 were related to non-translational aspects of gene expression and metabolic regulation such as transcription, splicing and phosphorylation. This may suggest that C3 genes act to regulate the decreased elongation levels of the housekeeping genes in C2 at the transcriptional and post-translational level. All enriched clusters and their corresponding gene annotations are detailed in Supplementary Table 7.

Seeking to determine whether similarity in the TDR of genes may be partially associated with the amino acid content of their proteins, we calculated the local pairwise alignment between every pair of genes within each cluster. Comparison of the alignment scores with the scores obtained for random group of genes of the same size, revealed a signal of AA similarity for clusters C1, C3, C5, and C9. These clusters turned out all to also be functionally enriched. The average score for these clusters was significantly higher than for random (*p* value < 0.01). This result suggests additional possible association between mutual elongation and protein functions.

To further show that changes in elongation rates encapsulate biological information, we calculated the correlations between the meiotic MTDR pattern of each pair of genes (MTDR co-score, Methods section). The obtained correlation revealed that genes associated with protein–protein interactions (PPI) tend to have more similar MTDR pattern than genes of non-interacting proteins (Spearman’s rank correlation of 0.206, *p* < 10^−323^, Fig. 3c). MTDR co-scores of interacting proteins turned out also to correlate with the PPI strength score (Fig. 3d), which represents the confidence level of the protein–protein interaction (Methods).^34^

### Positive regulation of the anaphase II pathway at the level of translation elongation, translation initiation and transcription

**Fig. 4:**
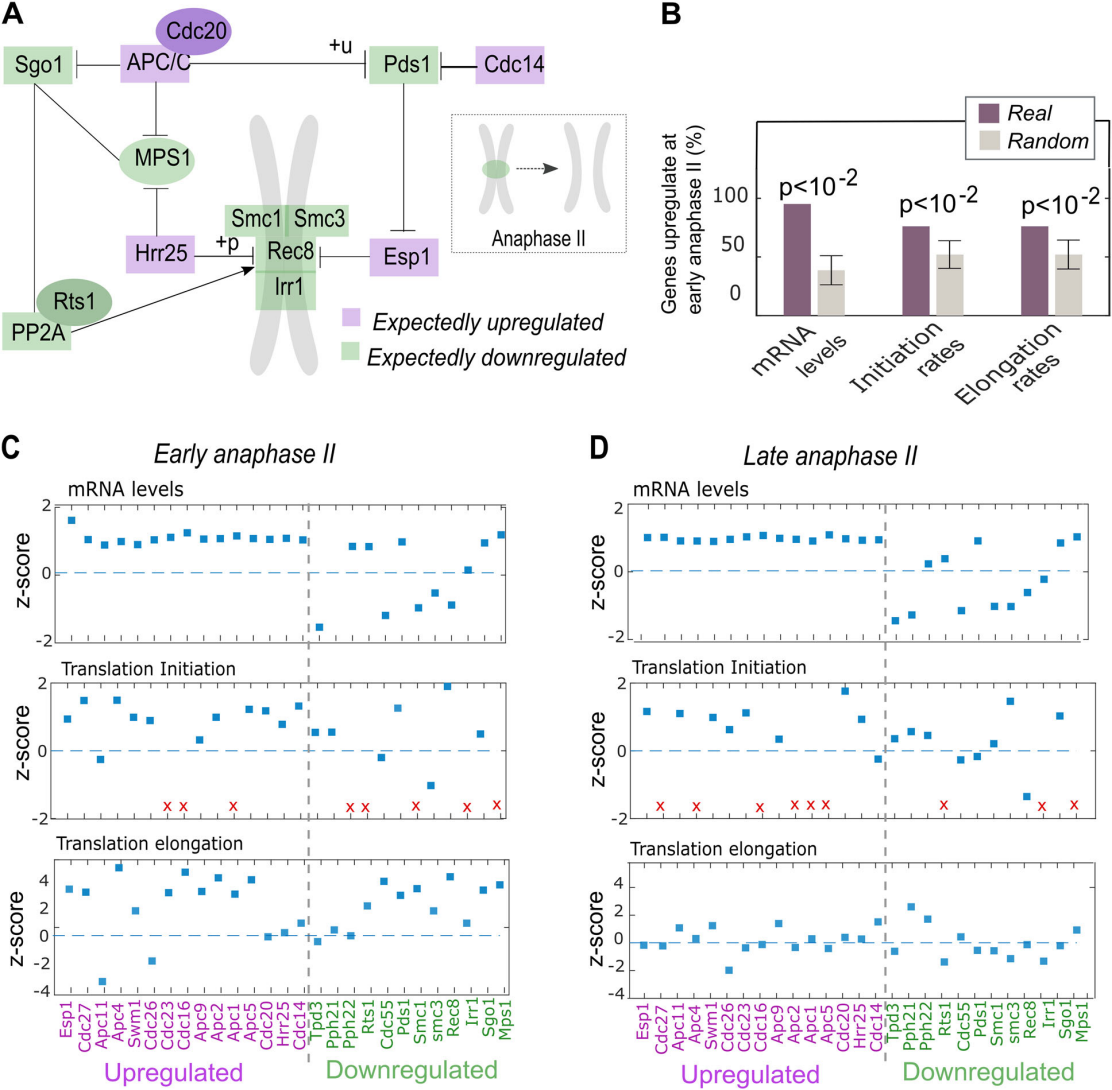
**a** Initiation of anaphase II occurs with the ubiquitination of the anaphase-promoting complex/cyclosome (APC/C). Once activated, the APC/C targets Securin (Pds1) for degradation, enabling the activation of separase (Esp1) which targets Rec8 for cleavage. Shugoshin protein (Sgo1) in complex with protein phosphatase 2A (PP2A) and its regulatory subunit Rts1 protect the cohesion until the onset of anaphase II.^88,89^ Hrr25 and Cdc14 contribute to the phosphorylation of Rec8 which is important in the cohesion cleavage.^80–82^ **b** The statistic describes the percentage of expected upregulated genes with increased rate at the onset of anaphase II (relatively to the average rate, z-score > 0). Gray bars represent the mean percentage based on 100 randomization, error bars represent the standard deviations (Methods). Corresponding p-values comparing the real and random numbers are denoted. **c**, **d** Relative rates (z-scores) of transcription, translation elongation and translation initiation for genes participating in early **c** and late **d** anaphase II. The expectedly upregulated genes (purple labels) are separated from the expectedly downregulated ones (green labels) by a vertical dashed line. Red x symbols denote genes for which IR could not be inferred

Anaphase II is the third stage of the second meiotic division in which the two sister chromatids of each chromosome separate and begin to move towards the opposite spindle pole body. Segregation of sister chromatids requires the removal of a stable cohesion complex that holds them at the centromeric region. Although the major cascade required for the removal of the cohesion in anaphase II is well established (depicted in Fig. 4a), the complete gene expression regulation at this time point has yet to be fully understood. Following the unique MTDR dynamics observed at the onset of anaphase II, we decided to further investigate the translational regulation at this time point. To this end, we compared rates of transcription, translation initiation and translation elongation for the genes involved or regulated in the anaphase II phase. Whereas elongation rates could be easily assessed for any given gene based on the MTDR, transcription and translation initiation rates (IR) were both dependent on the availability of ribo-seq and mRNA-seq data (see details in the Methods section). Within anaphase II genes, we distinguished between two groups: genes that are expected to undergo upregulation during anaphase II (i.e. promote the progression of anaphase II) and those expected to undergo downregulation at this time point (i.e. interfere with the progression of anaphase II, Methods). Remarkably, at the *onset* of anaphase II, the expected upregulated genes showed increased transcription and translation levels (all-*p* < 10^−2^, Fig. 4b). The expected downregulated genes on the other hand, did not show decreased expression rates (Supplementary Figure 4). However, since the cohesion is removed only after several processes including phosphorylation followed by cleavage of the Rec8 subunit, we speculated that the expression levels of genes acting to preserve the cohesion may decline only towards the end of anaphase II. To test this conjecture, we calculated the expression rates of the expected downregulated group also in a late time point during anaphase II (Methods). Indeed, some of the expected downregulated genes showed decreased expression rates only late at anaphase II (Fig. 4c, d). Rec8 for example, which holds the cohesion complex together and must undergo phosphorylation prior to being cleaved by separase, was found in high levels at the onset of anaphase II (Fig. 4c). However, at the late anaphase II, it decreased in all levels (Fig. 4d). Generally, whereas transcript levels exhibited a relatively homogenous pattern, MTDR and IR showed more subtle fluctuations (Fig. 4c, d).

### Ribosomal proteins exhibit low elongation rate at anaphase II but maintain high initiation rate during all meiosis stages

**Fig. 5:**
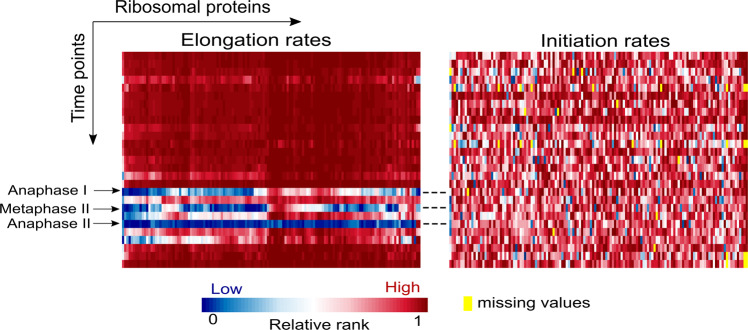
Translation elongation rates (left) and initiation rates (right) of the ribosomal proteins during meiosis. Each column represents a ribosomal protein and each row represents a time point along meiosis. Elongation rates are quantified by MTDR. Ribosomal MTDR and IR are represented by their rank among all genes such that blue and red entries represent low and high rates, respectively, relatively to the rates of all other genes. Yellow entries correspond to proteins for which initiation rates could not be inferred at specific time points (Methods)

Owing to their fundamental role in translation, ribosomal proteins undergo selection for efficient elongation that consumes less ribosomes and improves cellular fitness. However, at meiosis II, the most efficient codon usage is inversely correlated with the usage in other time points. Thus, their elongation rate is lower at this stage (Fig. 5, left). Despite the net decrease observed at the level of translation elongation at the end of anaphase I, at metaphase II and at anaphase II, the ribosomal proteins turned out to maintain relatively high IR in all time points, enabling translation to proceed during the different meiotic stages (Fig. 5, right).

## Discussion

Throughout the cell cycle, certain proteins need to be synthesized rapidly and in higher abundance to ensure the intact progression of each phase.^4,19,35,36^ In addition to other mechanisms such as transcriptional and posttranslational regulation, cells exert a tight control over mRNA translation. The ability to adjust translation rates for mRNAs presented in the cell in comparable abundance, enables a dynamic and rapid adaptation to sudden protein demand. Here we suggest that the elongation phase of translation also plays a functional and dynamic role in the regulation of mRNA translation during meiosis. Specifically, we show that changes in translation elongation efficiency during the yeast sporulation program ensure that transcripts of stage-specific proteins are selectively translated with higher efficiency. However, changes in translation elongation may contribute to the organism fitness not only through directly affecting the protein levels of genes. Among others, improved elongation rates can decrease error rate during translation,^37^ decrease traffic jams to promote better allocation of ribosomes,^38^ or affect mRNA degradation rate.^39,40^

The novelty of this study is reflected by the following three aspects: (1) The study is specifically focused on translational control at the elongation stage; (2) It provides a condition-specific view of elongation, differently from currently existing works which analyze elongation rates based on static/steady state conditions; (3) All *S. cerevisiae* genes are analyzed, including very lowly expressed genes which are harder to be ‘captured’ based on experiments only.

The decoding time of codons along the analyzed time points exhibited a dynamic, time point-specific pattern that could not be trivially explained by codon usage bias quantifications. The substantial changes in the decoding time of several codons from the highest to the lowest ranks, demonstrate a strong fine-tuning regulation of elongation in response to environmental changes. Particularly, the effect of rare codons on elongation regulation was found to be more condition-specific than the effect of frequent codons. At the amino acid level, the most extreme changes were observed for asparagine, which was found to have slow decoding rates during most stages of meiosis, but a relatively high decoding rate at the onset of anaphase II. Intriguingly, intracellular asparagine levels were found to play a critical role in cancer cell growth and proliferation.^41–43^ Furthermore, it was shown that low-asparagine diet could slow down the spread of breast cancer.^44^ Being the restrictive factor of the process, further limiting their levels in cancer cells may slow down the cancer growth rate. It is possible that mitosis and meiosis share some global translational/metabolic aspects and thus, amino acids that are found in limited levels during meiotic cell division (and hence, induce slow decoding rates) might also be limited during cancer cell growth.

Intriguingly, we found that Rec8 has increased elongation and initiation rates at the onset of anaphase II, although it was found in very low mRNA levels. It is possible that the efficient elongation may not be directly related to improved protein levels (despite the general correlation between these two variables). For example, it is possible that due to its functionality (*e.g*. various PPI with other proteins expressed at anaphase II), it undergoes selection for certain protein structures that are dictated by specific amino acids that induce the codons with the elevated elongation rates at this time point. However, although seemingly, Rec8 does not have to be synthesized at anaphase II, it is also possible that it has additional roles that can be further investigated. Indeed, several studies have raised the prospect for a cohesion formation in additional time points after the s-phase^45,46^ and suggested that its full function is not completely understood.^47^

Overall, the fluctuations in the codon elongation rates were, in most cases, compatible with those observed at the amino acid level. This may suggest that the shifts towards low TDR observed for certain codons, may be a consequent of slow amino acid metabolism or poor aminoacylation. It was indeed shown early on that the intracellular amino acid pool can change substantially under different growth conditions.^48^ Specifically, meiosis is initiated by an extracellular signal of deprivation in nitrogen and carbon,^33^ crucial components of all amino acids. Here we suggest that amino acids that were already limited in the pool prior to meiosis, have a greater effect on the decoding time of their codons. Other codon-related factors, such as the tRNA pool which can considerably change during the cell cycle, may also affect the actual TDR.^19^ Another potential cause for the changes in the TDR can be related to modification of the ribosome. Specifically, it is possible that changes in the ribosome during meiosis (*e.g*. via RNA modifications^49^) can affect the relative elongation speed of the different codons.

Today we understand that the elongation speed can affect protein folding during a co-translational folding process.^50,51^ However, measuring and modeling this process is very challenging and is currently assumed to be static^52^ (i.e. not to vary across different conditions). Thus, a potential future study can utilize the resultant changes in the elongation rates to study their relation to changes in the protein structure and folding, seeking to determine whether this relation can be condition-specific. The fact that the TDR pattern of interacting and non-interacting proteins turned out to be significantly different, provides additional and more general association between protein function and elongation speed. Such a relation can be explained, indirectly, based on the idea that follows: Genes associate with PPI are required to act together at the same time point and are thus expected to have similar protein abundance in the cell.^53^ To produce similar protein levels, these genes undergo similar translation and thus, have also similar elongation rates.

Importantly, the positive relation between translation rate and protein levels remains also when protein degradation is part of the regulation.^18^ However, incorporating large-scale measurements of degradation rates during meiosis can provide an interesting layer to the analyses. Nevertheless, measuring protein degradation remains a challenging task as for a given organism, proteomic data is usually available for approximately only half of the proteins (the most highly expressed,^54^). Thus, there is currently no approach to infer protein degradation rates for all proteins in all cell cycle steps.

Interestingly, at several time points in meiosis II, the estimated elongation rates were inversely correlated with those found in other time points. We suggest that housekeeping genes that are generally required for translation (such as ribosomal genes) undergo an evolutionary selection for codons optimally adapted to the standard/normal tRNA pool and thus, tend to be highly expressed at most time points along the cell cycle. Alternatively, genes required specifically only at several time points (such as anaphase II), have adopted non-optimal codon usage that would fit the unique tRNA pool presented in the cell at these time points. Nevertheless, the fact that a subset of genes is not regulated at the translation elongation level at a certain time point, does not imply that it does not regulated at all. It is plausible that the same genes are regulated at both anaphase I and II, but the regulation is not at the elongation level. Other types of regulation can be related, for instance, to global higher translation initiation at anaphase I, post-translational regulation, and protein or mRNA degradation. Considering transcript levels to determine the dynamic codon demand has revealed that rare codons are required significantly more than frequent codons during meiosis II. The idea that codon usage may induce cell cycle-dependent protein demand is supported by previous observation of enriched non-optimal codon usage in sets of cell-cycle-regulated genes.^19^ Here, however, we provide a high-resolution description regarding the way such codons regulate relevant pathways in meiosis. Since rare codons (which are usually slow) are expected to be the bottleneck of the translation rate along an mRNA, their modulation should have the largest effect on translation. Thus, an efficient way to control translation would be via elongation modulation of rare codons.

Improving the elongation speed of the rare codons for the genes that encode the proteins that are required at the onset of anaphase II is expected to improve their expression. It has been previously suggested that highly expressed mRNAs tend to include faster codons (that induce faster elongation) due to various reasons: First, faster codons lead to a direct increase in the translation rate and thereby, in the protein levels of their transcript.^55–61^ Second, ribosomes spend less time on faster codons. Thus, the usage of faster codons decreases the ribosomal density, which is specifically important for genes with many mRNAs. Faster codons on such genes should have a significant effect on minimizing the amount of energy required for translation via improving ribosomal allocation.^62–64^ Third, it is believed that the probability of translational errors and protein misfolding is generally reduced for faster codons.^37,61,65^ Since the negative effect of such errors is expected to be stronger for highly expressed genes, they are expected to undergo selection to include faster codons.

Since the cellular resources required for translation are limited, the translation of the large cluster of genes with fast elongation rates at the anaphase II, is expected to slow down the translation of other genes. Indeed, we observed a general translational repression at the elongation level at the onset of anaphase II. It has been already shown that during mitotic cellular division, global translation is inhibited at the 5’ cap-dependent initiation level^66–68^ so the ribosome accesses mRNAs independently of the cap-binding protein eIF4E using a *cis*-regulatory element known as the internal ribosome entry site (IRES).^12,69^ It was also shown that during mitosis translation is globally arrested at the elongation level.^70^ Here we suggest that translational repression at the elongation level also occurs at the onset of anaphase II, in line with the fact that the second meiotic division resembles mitosis in a manner that similar forces and attachments operate in both.^71^ Despite a net decrease in translation elongation at the onset of anaphase II, the translation initiation rates of the ribosomal proteins turned out to be maintained at relatively (to other genes in these conditions) high levels. It is thus plausible to suggest that cells exert a mechanism to maintain high initiation rates for the ribosomal proteins to compensate their reduced elongation due to non-optimized codons.

While several recent studies suggested that elongation may contribute to translation regulation,^72^ our findings demonstrated a high-resolution functional and dynamic role for translation elongation in translational control under different developmental and cellular conditions. The codon-based calculation of the MTDR allowed us to perform a genome-wide analysis that include very lowly expressed genes that otherwise could not be analyzed based on direct ribosome profiling measurements. Along with a better understanding of evolutionary constraints and novel insights into gene expression regulation during meiosis, this work also provides a practical implication on gene expression engineering by which translation elongation should be an important consideration in the engineering of any intracellular system that involves gene expression optimization.

## Methods

### Ribo-Seq data

Ribosome footprints and mRNA-seq sampled through the 27 time points in^4^ were retrieved from https://www.ncbi.nlm.nih.gov/geo/ with series accession number GSE34082. Reads of both, footprints and RNA, were mapped according to the mapping approach described in.^21^

### MTDR calculation

Codon decoding rates were calculated according to the stages described in.^23,24^ As in,^23^ the first and last 20 codons were excluded from the per-gene NFC profiles in order to account for potential biases related to these regions. The coverage (in percent) for each profile was calculated based on the filtered profiles and only genes with a coverage of at least 40% were included in the subset. In addition, the minimal number of occurrences for each codon was set to 100. Our results, however, remained robust also for small changes in these thresholds. Based on this subset of genes, per-codon NFC distributions were generated. As been shown in,^24^ the NFC distribution of a codon can be represented by an EMG distribution, a superposition of two distributions: a normal distribution characterized by mean μ and variance *σ*^2^, and a negative exponential distribution with rate parameter λ. While the normal component describes the typical decoding time of the codon, the exponential component describes the non-typical decoding time. The non-typical component can be a result of noise and biases, or may be related to relatively rare phenomena of translational pauses and ribosomal interactions such as traffic jams. Although these rare phenomena can be of biologically meaningful, the exponential component is filtered since the non-typical rates it represents, are not suitable for estimating elongation in genes that do not have ribo-seq measurements. The parameters μ, *σ*^2^, and λ were estimated by fitting the measured NFC distributions to the EMG distribution, under the log-likelihood criterion.^23^ The typical decoding time of a codon was then determined by the mean of the normal distribution (*μ*) and the typical decoding rate of a codon was defined accordingly by 1/*μ*. Although the TDR can be influenced also by secondary structures along the mRNA which are expected to slow down elongation,^59^ this aspect cannot be fully taken into account in our codon-centric analysis since the current models for predicting mRNA folding are based on local RNA segments, rather than on a single codon. In addition, these models are based on static sequence features, whereas our approach is focused on dynamic aspects. Nevertheless, it is possible that future techniques to measure dynamic mRNA structures generated along the transcript will allow the consideration of mRNA folding in dynamic decoding rates analyses. Outliers were removed from the NFC distribution of each codon at each time point in the following way: let *NFC*_*i*_ denote the *i*-th point in the codon NFC distribution and *n*_*i*_ be the number of points in its distribution. We calculated the probability (*p*_*i*_) to see a value larger or equal to *NFC*_*i*_ based on the probability density function fitted to the codon (EMG distribution) and removed points for which *p*_i_* *n*_*i*_ was lower than 0.001.

### TDR versus the median of the NFC distribution

The typical elongation rates estimated by the TDR approach were compared to elongation rates estimated by the median of the NFC distribution. In general, the median-based results were less significant, however, in agreement with the original TDR-based results. The average Spearman’s rank correlation between the decoding rates obtained by the approach (TDR) and the median-based rates was 0.436, for all time point with a minimal RC coverage of 10% (Coverage distribution is presented in Fig. 1b). In addition, clustering of the median-based genes’ decoding rates along meiosis revealed some overlapping clusters. Particularly, the clusters that included highly expressed genes at most time point were the most robust clusters. For example, the TDR-based cluster C2 presented in Fig. 3a, which turned out to be composed of translation-related genes, had a corresponding median RC-based cluster with an overlap of 496 genes (89%); the dominant GO term ‘cytoplasmic translation’ was also included with a slightly higher p-value (7*10^−84^, compared to 1.7*10^−86^ in the original cluster). More specific GO terms such as branched-chain amino acid biosynthetic process (p-value increased from 1.1*10^−10^ to 2.9*10^−6^) and vitamin binding (p-value has only slightly changed, from 1.5*10^−6^ to 1.3*10^−6^) were also observed. For clusters containing mainly lowly expressed genes, the signal was much weaker, yet, in agreement with the original cluster. For example, the TDR-based cluster C1 presented in Fig. 3a which included functions related to the M phase and anaphase II, had a corresponding cluster with 391 (63%) genes in common; these genes turned out also to be enriched with functions related to M phase (corresponding *p-*value increased from 1.1 × 10^−27^ in the original TDR-based cluster, to 7.9 × 10^−10^).

### MTDR standardized score

To enable the comparison of MTDR between different time points while controlling for different coverage and biases in the different time points, MTDR values were standardized to have the same mean in all time points. Specifically, for each time point, mean MTDR and standard deviation were calculated based on all genes and z-scores were obtained for each gene by taking the difference between its MTDR and the mean, divided by the standard deviation.

### Codon adaptation groups

For the analysis described in Fig. 2, two groups of codons were defined based on the per-codon CAI reported in^32^ (See also Supplementary Table 2). For each amino acid, we chose the codon with the maximal and minimal CAI to construct the ‘High CAI’ and ‘Low CAI’ groups, respectively. In order not to bias the results by including codons with no synonymous counterparts, we filtered the two codons of Methionine and Tryptophan.

### tRNA Adaptation Index

The tAI of each codon was calculated based on the relative adaptiveness of each codon as in.^31^ The tRNA copy number of each tRNA was retrieved from the genomic tRNA database at http://gtrnadb.ucsc.edu/ for *Saccharomyces cerevisiae s288c*. The tAI values used here are provided in Supplementary Table 3.

### Coefficient of Variation in TDR

For each time point, codons’ TDR were ranked from 1 to 61 (where 1 denotes the lowest TDR and 61 denotes the highest). Then, CV of the ranked TDR was calculated for each codon using the following formula:

$${\rm{CV}} = \left( {\frac{{{\rm{S}}\_{\rm{TDR}}}}{{{\rm{M}}\_{\rm{TDR}}}}} \right) \cdot 100$$

Where *M_TDR* and *S_TDR* are the mean and standard deviation of the ranks of the codon along the analyzed time points, respectively. The CV calculated for the amino acids was performed in the same way.

### Quantifying the dynamic demand for each codon

The genomic per-time point demand for each codon was calculated based on the frequency of the codon in all transcripts. Specifically, the number of occurrences of the codon in the gene was multiplied by the transcript level of the gene (RNA-seq in RPKM^4^) and the product was summed over all genes. The total frequency of each codon in all transcripts was normalized by the total frequency of the corresponding amino acid and is provided as Supplementary Table 4. Transcript levels are provided as Supplementary Table 5.

### Per-amino acid Average TDR

To estimate the per-time point TDR of each amino acid, we calculated *TDR*_*AA*_, a weighted average based on the TDR of its synonymous codons in the following way:

$${\rm{TDR}}_{AA} = \mathop {\sum }\limits_{i = 1}^n w_i \cdot {\rm{TDR}}_i$$

Where *n* is the number of synonymous codons, *w*_*i*_ is the normalized genomic frequency of the *i*-th codon (i.e., the number of occurrences of the *i*-th codon normalized by the total number of occurrences of the amino acid) and *TDR*_*i*_ is the per-time point TDR of the *i*-th codon.

### Clustering and functional enrichment analysis

Unsupervised clustering of genes’ MTDR (z-scores) was performed by the CLICK algorithm^73^ via the EXPANDER tool version 7.1.^74^ Functional enrichment analysis on the resultant clusters was performed by the TANGO tool of EXPANDER. Paralogous gene sequences were excluded from the clustering analysis as they share the same sequence and thus, the same MTDR.

### MTDR co-score

The co-score given to each pair of genes was calculated by:

$${\rm{MTDR}}\,co - {\rm{score}}_{i,j} = r\left( {\left[ {tp} \right]_i,\left[ {tp} \right]_j} \right)$$

where [*tp*]_*i*_ is the vector of MTDR values calculated for the *i*-th gene in all time points and *r* is the Pearson correlation between the two MTDR vectors of the *i*-th and the *j*-th gene.

### The effect of the C1 genes on translation using the whole cell simulation of translation

In order to examine the role of the unique elongation pattern of the C1 genes (Fig. 3) on translation at the anaphase II onset, we utilized the whole cell simulation of translation that includes all the ribosomes and mRNAs in the cell with parameters inferred based on ribo-seq data, to allow a good reflection of in vivo translation.^75^ According to this model, ribosomes from the free pool are allocated to the different transcripts presented in a cell at a certain time point based on the initiation rates of the genes. After a ribosome is entered, it progresses along the codons in a speed that is determined by the elongation rate of each codon. A ribosome that encounters a stop codon, completes translation and re-joins the free pool (Supplementary Figure 3A). For the simulation, we set the total number of mRNAs in the cell to 60,000, based on the number reported in.^76^ As the number of ribosomes is of the order of 2*10^5^ in a *S. cerevisiae* cell,^77^ we set the total pool of ribosomes to 200,000.^77^ A chuck size of 10 codons was used to occupy a ribosome based on the simulation in.^75^ We used the initiation rates inferred here, and the TDR at the anaphase II onset as the estimated per-codon elongation rates. To run the model at anaphase II, we first mapped the TDR at the exponential time point to the decoding rates used in^63^ and the initiation rates at the exponential time point to those used in^75^ using a linear interpolation. Then, we used the mapping on the TDR and initiation rates on anaphase II. To test the effect of synonymous changes of genes in the C1 cluster on the pool of ribosomes, we generated 20 versions of a randomized genome by changing the coding sequences of the genes in the C1 cluster in the following way: for each amino acid in each gene, we have randomly drawn a synonymous codon from a distribution that represents the original codon demand (that is, the genomic codon usage bias of the genome multiplied by the number of transcripts that require it at normal vegetative growth condition). We found that for simulation with the original C1 codons, the pool included 60,000 ribosomes while for the second case (random codons based on the genomic distribution of codons) the average free pool over all randomizations was decreased by 5.83% (z-score = 24.37). The size of the free ribosomal pool is directly related to translation initiation efficiency which directly, and usually linearly, affects translation rate. Indeed, the mean estimated translation rate (over all genes) decreased on average in 6.04% (z-score = 36.65) for the randomized genomes (Supplementary Figure 3B). Since ~80% of the intracellular energy is spent on translation,^78^ an increase of 6% in the free ribosomal pool should have a similar effect on increasing the growth rate; this is a *very significant* effect in case of micro-organisms.

### Construction of the anaphase II pathway

The anaphase II pathway genes described in Fig. 4 were collected from several resources including the budding yeast meiosis pathway from the Kyoto Encyclopedia of Genes and Genomes (KEGG) pathway database^79^ and additional information manually curated from recent papers. Specifically, we included casein kinase (Hrr25) which has been very recently shown to contribute to the phosphorylation of centromeric Rec8 at anaphase II.^80^ Also, based on the findings of Attner and Amon^81^ which have also been experimentally supported by,^82^ we added the Cdc Fourteen protein that was found to contribute to the metaphase to anaphase transition also in meiosis II. We removed the KEGG protein Ama1 which was found to be required for the first meiosis and spore formation but not for the second meiosis.^83^

### The expectedly up/down regulated genes during anaphase II

Based on KEGG^79^ and the additional papers used for the pathway construction^80–82^, and as reviewed in,^84^ we classified the genes participating in the anaphase II stage of the yeast meiosis based on their expected direction of regulation at anaphase II. During anaphase II the centromeric cohesion which holds sister chromatid together is removed and sister chromatids are segregated. Thus, genes whose products are expected to promote the removal of the cohesion were classified as ‘expectedly upregulated genes’, and genes whose products act to maintain the cohesion, or constitute its components, were classified as ‘expectedly downregulated genes’. Due to significantly low RC and RNA-seq coverage, we excluded the Doc1 protein, a member of the APC/C complex. Full lists of these genes are presented in Supplementary Tables 9-10.

### Definition of early and late anaphase II

We used the time points classifications in^4^ to confine the anaphase II stage. Based on the classification, three time points fall between the stage of anaphase II (labeled consecutively as 11, 12 and 13). For the analysis described in Fig. 4, we set the ‘11’ time point to denote ‘early anaphase’ and the ‘13’ time point to denote ‘late anaphase’.

### Inference of translation initiation rates

To infer initiation rates during meiosis we implemented an optimization approach using a stochastic computational model that simulates translation, the TASEP.^85^ According to the TASEP model of translation, the translation rate of a given gene (along with the number of ribosomes on its mRNA and other translational-related features) is evaluated based on inputted initiation rate and the local translation rates of its codons. Here, we implemented a backward approach by which we determined the desired TASEP output and searched for the input initiation rate that would lead to the closest output. Based on our approach, the initial initiation rate is guessed and iteratively changed to fit a predicted output number of ribosomes. Specifically, we used ribosome occupancy data from^86^ to get the expected number of ribosomes and the ribosomal density on each mRNA under vegetative growth condition. We calculated the correlation between ribosome occupancy and the analyzed RC data to verify compatibility (r = 0.4615, *p* = 1.17*10^−275^, Supplementary Figure 5). Then, we calculated ribosomal density in reads per kilobase million (RPKM) based on the ribosome profiling data sampled also at a time point during vegetative growth^4^ (Supplementary Table 8). In the next step, we performed a linear interpolation to map ribo-seq footprints average (RPKM) at each time point into the expected number of ribosomes, which is one of the TASEP outputs. Finally, we inputted the estimated codons’ TDR and an initial IR guess into the TASEP, and performed a binary search to gradually change the IR such that the number of ribosomes on the mRNA predicted by the TASEP fits the expected number of ribosomes. The output of this procedure is an estimated initiation rate for each mRNA. As this approach requires ribosomal density calculations, we were not able to infer time point-based initiation rates for genes that did not produce a RC profile at the time point in question.

### Statistical analysis of expression rates during anaphase II

The analysis described in Fig. 4 panels B-D was aimed at quantifying the tendency of the upregulated group to increase early at anaphase II, and the tendency of the downregulated group to decrease late at anaphase II. The statistical test was performed in the following way: First, for each gene within the expectedly upregulated group and for each tested expression level (transcription, translation initiation and elongation), we calculated a z-score quantifying the distance in standard deviations between the rate at the onset of anaphase II and the average rate over all time points.

### PPI data

PPI network and confidence scores were downloaded from the STRING database version 10.5.^34,87^ For the PPI analyses, we used only proteins for which the interaction type (as defined by STRING) is of type ‘binding’.

### Controlling for variability in the number of NFC per codon

The per-codon NFC distributions used for the TDR calculation, are generated based on the occurrences of each codon in the reference set of genes used at a given time point. Owing to their number of occurrences in the genome, frequently used codons tend naturally to include more points in their NFC histograms. To validate that the findings presented in Fig. 2d are not biased by variability in size of the NFC distribution, we sampled the same number of RC for all codons at a given time point and repeated the statistical analyses. Specifically, the chosen number of RC was dictated separately for each time point, by the maximum between 100 and the minimal number of occurrences of a codon at that time point. Importantly, all the statistical tests reported here were robust to this control.

### Reporting Summary

Further information on experimental design is available in the Nature Research Reporting Summary linked to this article.

## Supplementary information

Reporting Summary

Supplementary results

suppl. Table 1

suppl. Table 4

suppl. Table 5

suppl. Table 6

suppl. Table 7

suppl. Table 8

## Data Availability

The authors declare that the main data supporting the findings of this study are available within the article and its Supplementary Information files. Extra data are available from the corresponding author upon request.

## Supplementary information

**Supplementary information** accompanies the paper on the *npj Systems Biology and Applications* website (10.1038/s41540-019-0089-0).
