# Supplementary material for: Novel insights into gene expression regulation during meiosis revealed by translation elongation dynamics
Source: NPJ Syst Biol Appl. 2019 Apr 4;5:12. doi: 10.1038/s41540-019-0089-0 (PMC6449359; doi:10.1038/s41540-019-0089-0)
Supplement: Supplementary file 2 — Supplementary results [file 41540_2019_89_MOESM2_ESM.pdf]

Supporting material for

Novel Insights into Gene Expression Regulation during Meiosis Revealed by Translation Elongation Dynamics

Renana Sabi[1] and Tamir Tuller[1,2*]

The file includes supplementary figures and short tables.

Tables longer than one page are provided in Excel format. Captions appear at the end of the file.

1 Department of Biomedical Engineering, Tel Aviv University, Tel Aviv, Israel.
2 The Sagol School of Neuroscience, Tel-Aviv University, Tel-Aviv, Israel.
* To whom correspondence should be addressed tamirtul@post.tau.ac.il

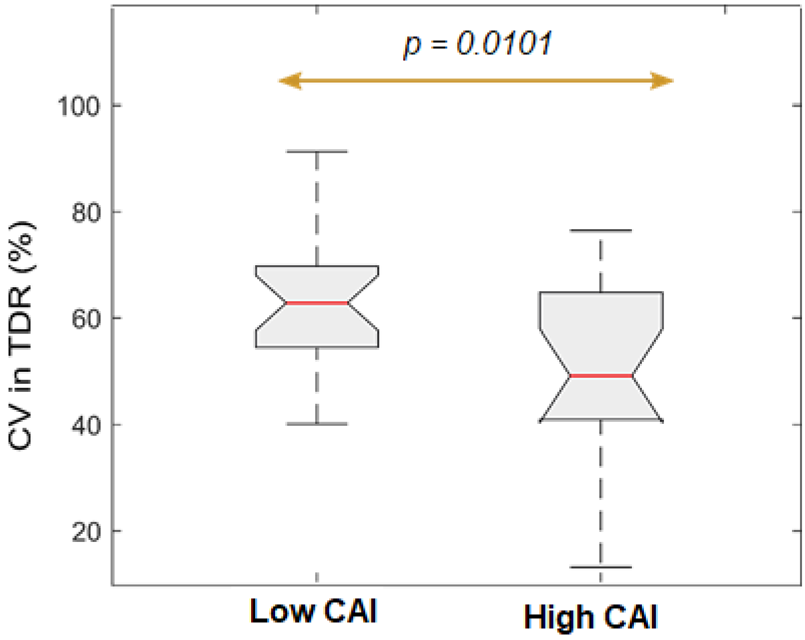

Figure1 . Robustness analysis for Figure 2D. The same number of read counts were sampled for all codons at each time point, resulting in an NFC distribution of the same length for all codons. Based on these Distribution, per-codon TDR were inferred, and the coefficient of variance in TDR was calculated. Two groups of codons were compared: frequent (very high CAI) and rare (very low CAI). Medians are marked by horizontal red lines. P-value corresponding to the statistical difference between the medians based on a right-tailed Wilcoxon rank sum test is denoted above.

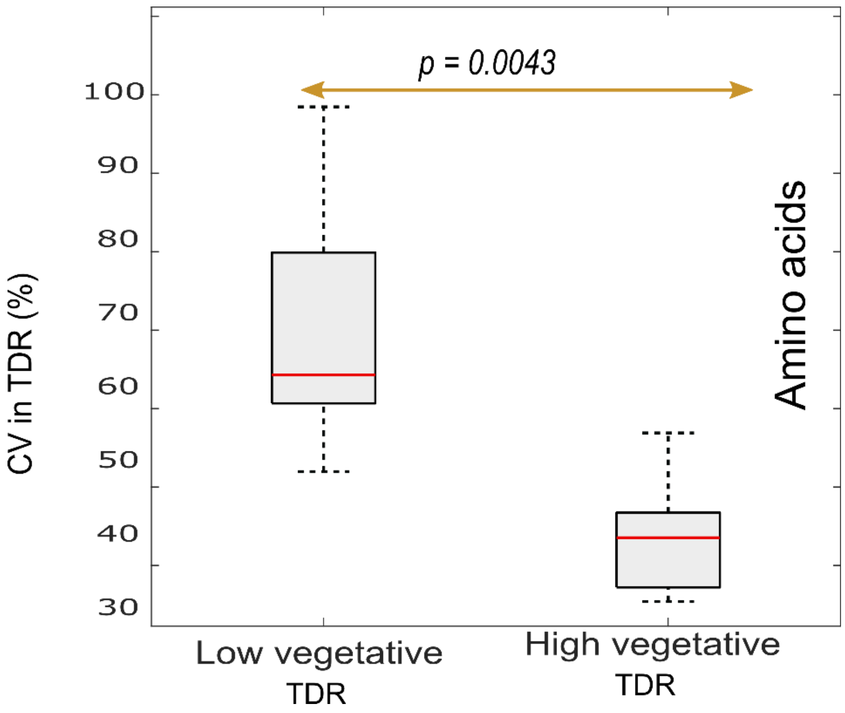

Figure 2. Robustness analysis for figure 2F. The same number of read counts were sampled for all codons at each time point, resulting in an NFC distribution of the same length for all codons. The CV in TDR of amino acids with slow and high TDR at the vegetative growth time point was recalculated and compared (Methods). Medians are marked by horizontal red lines. P-value corresponding to the statistical difference between the medians is denoted.

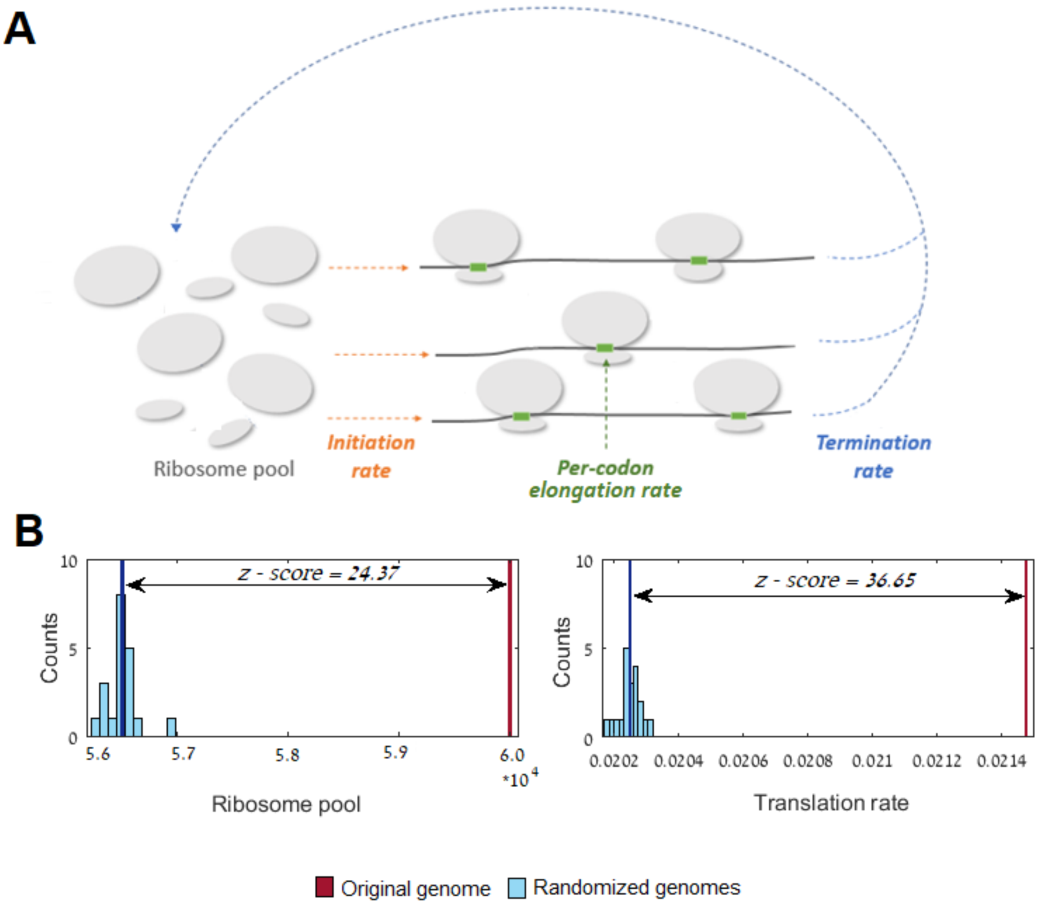

Figure 3. A. General illustration of whole cell translation simulation. The whole cell simulation of translation includes all ribosomes and all mRNAs in the cell such that each mRNA is a lattice of codons (the parameters used here are in the Methods). Ribosomes from the free pool (gray) enter each mRNA according to its initiation rate and then, ribosome progress on each codon according to its specific elongation rate which is inferred here based on the TDR (green). The pool is changed dynamically such that ribosomes the complete translating the mRNA are added to the free pool of ribosomes (termination rates are in blue), and ribosomes that start translating an mRNA are subtracted from the free pool of ribosomes (initiation rates are in orange). **B. Decrease in translation following synonymous changes in C1 genes.** Changes in the pool of free ribosomes and the average translation rate as obtained by simulating translation with whole cell simulation [1]. The original values obtained by the model with the original sequences at the anaphase II onset are shown in red bars. The distribution of the values obtained by the model with the genome that includes randomized C1 sequences are shown in light blue, the average values are shown in blue lines. random genome as obtained by simulating translation with the model that includes a finite pool [1]. Z-scores corresponding to the significant distance between the real and the average random values are presented above a double arrow.

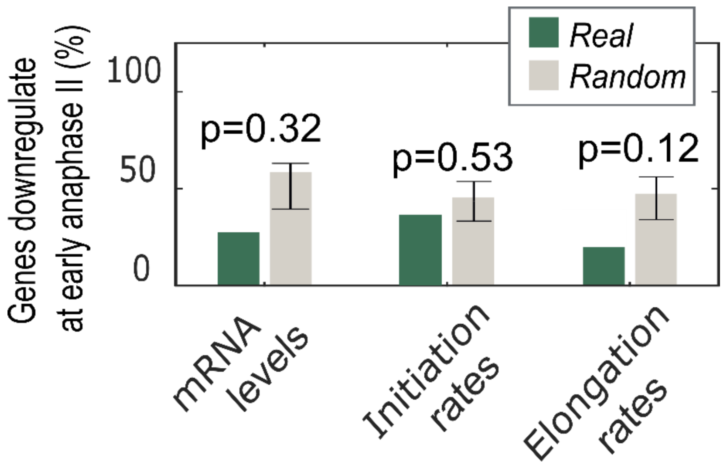

Figure 4. Expression rates of expectedly regulated genes at anaphase II. A complementary analysis of Figure 4B by which the expectedly down regulated genes do not show a signal of negative regulation at early anaphase II at the level of transcription, translation initiation and translation elongation. The statistics describes the percentage of expectedly downregulated genes with decreased rate at the onset of anaphase II (relatively to the average rate in all time point, z-score > 0). Gray bars represent the mean percentage based on 100 randomization, error bars represent the standard deviations (Methods). Corresponding p-values comparing the real and random numbers are denoted.

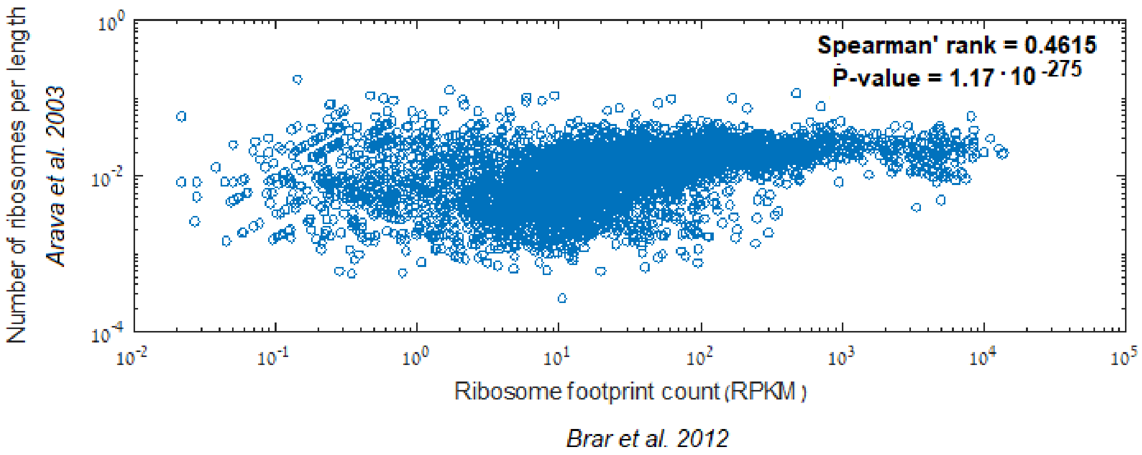

Figure 5. Ribosome profiling versus ribosome occupancy data. Dot plot of the ribosome profiling data by Brar *et al.* [2] at the exponential time point versus the ribosome occupancy data by Arava *et al.* [3]. To make both measures represent densities, the average number of ribosomes was normalized by the length of the gene in codons. Axes are presented in log scale. Spearman's rank correlation and the corresponding p-value appear to the right.

Codon	CAI	Codon	CAI
TTT	0.113	ATA	0.003
TTC	1	ATG	1
TTA	0.117	ACT	0.921
TTG	1	ACC	1
TCT	1	ACA	0.012
TCC	0.693	ACG	0.006
TCA	0.036	AAT	0.053
TCG	0.005	AAC	1
TAT	0.071	AAA	0.135
TAC	1	AAG	1
TGT	1	AGT	0.021
TGC	0.077	AGC	0.031
TGG	1	AGA	1
CTT	0.006	AGG	0.003
CTC	0.003	GTT	1
CTA	0.039	GTC	0.831
CTG	0.003	GTA	0.002
CCT	0.047	GTG	0.018
CCC	0.009	GCT	1
CCA	1	GCC	0.316
CCG	0.002	GCA	0.015
CAT	0.24	GCG	0.001
CAC	1	GAT	0.554
CAA	1	GAC	1
CAG	0.007	GAA	1
CGT	0.137	GAG	0.016
CGC	0.002	GGT	1
CGA	0.002	GGC	0.02
CGG	0.002	GGA	0.002
ATT	0.823	GGG	0.004
ATC	1		

Supplementary Table 2. Codon Adaptation Index in yeast. Values for the 61 sense codons are based on the calculations in [4]. For each amino acid, the CAI is calculated relatively to all synonymous codons, thus, a CAI value of 1 represent the most adapted codon per-amino acids.

Codon	tAI	Codon	tAI
TTT	0.363	ATA	0.123
TTC	0.615	ATG	0.615
TTA	0.431	ACT	0.677
TTG	0.753	ACC	0.487
TCT	0.677	ACA	0.246
TCC	0.487	ACG	0.14
TCA	0.184	AAT	0.363
TCG	0.12	AAC	0.615
TAT	0.29	AAA	0.431
TAC	0.492	AAG	1
TGT	0.145	AGT	0.072
TGC	0.246	AGC	0.123
TGG	0.369	AGA	0.677
CTT	0.036	AGG	0.278
CTC	0.061	GTT	0.862
CTA	0.184	GTC	0.62
CTG	0.059	GTA	0.123
CCT	0.123	GTG	0.162
CCC	0.088	GCT	0.677
CCA	0.615	GCC	0.487
CCG	0.197	GCA	0.307
CAT	0.254	GCG	0.098
CAC	0.431	GAT	0.581
CAA	0.554	GAC	0.985
CAG	0.239	GAA	0.862
CGT	0.369	GAG	0.399
CGC	0.266	GGT	0.581
CGA	0.000036	GGC	0.985
CGG	0.061	GGA	0.184
ATT	0.8004	GGG	0.182
ATC	0.576		

Supplementary Table 3. Per-codon tRNA Adaptation Index. Values for the 61 sense codons are calculated as presented in [5]. tRNA copy numbers used for the calculation were downloaded from the Genomic tRNA database [6,7].

Systematic Protein Name	Systematic Gene Name
**Esp1**	YGR098C
**Cdc27**	YBL084C
**APC11**	YDL008W
**APC4**	YDR118W
**Swm1**	YDR260C
**Cdc26**	YFR036W
**Doc1***	YGL240W
**Cdc23**	YHR166C
**Cdc16**	YKL022C
**APC9**	YLR102C
**APC2**	YLR127C
**APC1**	YNL172W
**APC5**	YOR249C
**Cdc20**	YGL116W
**Ama1**	YGR225W
**Hrr25**	YPL204W
**Cdc14**	YFR028C

* Doc1 was removed from the analyses due to low coverage of RC and RNA-seq.

Supplementary Table 9. List of the expectedly up regulated genes at anaphase II. Proteins in the list are expected to promote the removal of the cohesion. Details on gathering the list are in the Methods section.

Systematic Protein Name	Systematic Gene Name
Tpd3	YAL016W
Pph21	YDL134C
Pph22	YDL188C
Rts1	YOR014W
Cdc55	YGL190C
Pds1	YDR113C
Smc1	YFL008W
Smc3	YJL074C
Rec8	YPR007C
Irr1	YIL026C
Sgo1	YOR073W
Mps1	YDL028C

Supplementary Table 10. List of the expectedly down regulated genes at anaphase II. Proteins in the list constitute components of the cohesion complex that holds sister chromatids together or acting to maintain it. Details on gathering the list are in the Methods section.

Captions of Excel Tables

Supplementary Table 1. TDR of the codons at each of the time points. Rows represent codons and columns represent time points (ordered by sporulation progression, as determined by [2]). TDR are normalized by the average TDR of the corresponding time point.

Supplementary Table 4. The total frequency of each codon in all transcripts at each time point (Methods) used to quantify the dynamic demand for each codon. Rows represent codons and columns represent time points. Values for each codon are normalized by the total frequency of the corresponding amino acid.

Supplementary Table 5. Transcript levels along the time points. Quantified for each gene by mapping the RNA-seq data of [2] (Methods) and calculating RPKM. Rows represent genes and columns represent time points.

Supplementary Table 6. Per-gene MTDR along the time points. Rows represent genes and columns represent time points. MTDR are normalized by the average MTDR of the corresponding time point.

Supplementary Table 7. Clustering of the genes based on MTDR. The first sheet includes the gene annotations enriched in each cluster and the corresponding p-value and the second sheet include the list of genes within each enriched cluster.

Supplementary Table 8. Ribosomal density along the time points. Quantified for each gene by mapping the Ribo-seq data of [2] (Methods) and calculating RPKM. Rows represent genes and columns represent time points.

1. Zarai, Y. & Tuller, T. Computational analysis of the oscillatory behavior at the translation level induced by mRNA levels oscillations due to finite intracellular resources. *PLOS Comput. Biol.* **14,** e1006055 (2018).
2. Brar, G. A. *et al.* High-Resolution View of the Yeast Meiotic Program Revealed by Ribosome Profiling. *Science (80-.).* **335,** 552–557 (2012).
3. Arava, Y. *et al.* Genome-wide analysis of mRNA translation profiles in Saccharomyces cerevisiae. *Proc. Natl. Acad. Sci.* **100,** 3889–3894 (2003).
4. Sharp, P. M. & Li, W. H. The codon Adaptation Index--a measure of directional synonymous codon usage bias, and its potential applications. *Nucleic Acids Res.* **15,** 1281–95 (1987).
5. dos Reis, M., Savva, R. & Wernisch, L. Solving the riddle of codon usage preferences: a test for translational selection. *Nucleic Acids Res.* **32,** 5036–44 (2004).
6. Chan, P. P. & Lowe, T. M. GtRNAdb: a database of transfer RNA genes detected in genomic sequence. *Nucleic Acids Res.* **37,** D93–D97 (2009).
7. Chan, P. P. & Lowe, T. M. GtRNAdb 2.0: an expanded database of transfer RNA genes identified in complete and draft genomes. *Nucleic Acids Res.* **44,** D184–D189 (2016).
